# Acquired hypoprolactinemia in men, possible phenotype

**DOI:** 10.1007/s11154-024-09895-9

**Published:** 2024-07-27

**Authors:** Giovanni Corona, Giulia Rastrelli, Clotilde Sparano, Linda Vignozzi, Mario Maggi

**Affiliations:** 1 Endocrinology Unit, Azienda-AUSL Bologna, Bologna, Italy; 2 Female Endocrinology and Gender Incongruence Unit, Mario Serio Department of Experimental and Clinical Biomedical Sciences, University of Florence, Florence, Italy; 3 Endocrinology Unit, Mario Serio Department of Experimental and Clinical Biomedical Sciences, University of Florence, Viale Pieraccini 6, Florence, 50139 Italy

**Keywords:** Prolactine, Hypoprolactinemia, Diabetes mellitus, Metabolic sydrome, Erectile dysfunction, Sexual dysfunction

## Abstract

The physiological role of prolactin (PRL) in men is still not well defined. The pathological increase is characterized by sexual function impairment along with possible negative consequences in body composition and metabolic profile. Conversely, the clinical significance of reduced PRL levels was only partially investigated or mainly neglected. The present paper aims to summarize and critically discuss possible phenotypes characterizing male subjects with reduced PRL levels. When possible, meta-analytic results were provided. Available data derived from patients seeking medical care for sexual dysfunction as well as from cross-sectional and longitudinal studies showed that low PRL in males is associated with a worse metabolic phenotype (including diabetes mellitus), mood disturbances (including anxiety and depression), and sexual dysfunctions (including psychogenic erectile and ejaculatory dysfunctions). Whether or not these features are direct consequences of reduced PRL levels or whether the latter reflect other pathway impairments such as serotoninergic failure cannot be clarified. The present data, however, emphasize that a deficiency of PRL should be taken into account and need further investigations.

**Supplementary Information:**

The online version contains supplementary material available at 10.1007/s11154-024-09895-9.

## Introduction

Prolactin (PRL) is a pleiotropic 23 KDa polypeptide discovered in the early thirties of the last century and produced by many cells throughout the human body, but mainly secreted in the bloodstream from the anterior pituitary [1]. It serves many biological functions, but its main role in mammals is to favor milk production by controlling mammary gland development (mammogenesis), the onset of lactation (lactogenesis), and galactopoiesis [2]. The PRL receptor (PRLR) is a single-pass transmembrane receptor belonging to the cytokine receptor superfamily, acting through Janus Kinase (JAK) and Signal Transducer and Activator of Transcription 5 (STAT5). Although its function in women is well established, the physiological role of PRL in men is still unknown. Unlike other pituitary hormones or hormones from other endocrine glands, a clinical condition characterized by an isolated deficiency of PRL has been scarcely investigated, even in women. Recently, three cases of isolated PRL deficiency have been described in female subjects from one family with post-partum alactogenesis due to a PRL gene mutation [3]. No other phenotype was apparent, and fertility, along with normal menstrual cycling, was preserved [3]. The latter finding further corroborates the essential role of PRL in milk production. Recently, several lines of evidence derived from clinical studies, reviewed elsewhere [4–6], recognize an ancillary role of PRL as a metabolic hormone involved in supporting and storing the required substances to favor mammogenesis, lactogenesis, and galactopoiesis during pregnancy and breastfeeding. Pathological excess of PRL in women mimics the scenario of pregnancy and lactation with oligomenorrhea/amenorrhea and galactorrhea, along with hypogonadotropic hypogonadism. Other metabolic correlates of hyperprolactinemia are obesity, hyperinsulinemia and insulin resistance, dyslipidemia, and altered lipolysis [4–6]. Accordingly, hyperprolactinemia was associated with an increased risk of cardiovascular (CV) and overall mortality [7], as also substantiated in a recent meta-analysis [8]. However, in men, clinical symptoms of hyperprolactinemia are scanty and mostly related to hypogonadotropic hypogonadism and sexual dysfunctions, with hypoactive sexual desire (HSD) and erectile dysfunction (ED) being the most specific correlates, as demonstrated also by a recent meta-analysis [9]. In fact, treatment of hyperprolactinemia reverted the sexual complaints [9].

Fifteen years ago, we originally described a male phenotype of hypoprolactinemia in a cohort of 2,531 men consulting for sexual dysfunction selected for being without hyperprolactinemia (PRL > 35 ng/mL) or pituitary disorders [10]. The phenotype we described included increased sexual dysfunctions and a worse metabolic and psychological functioning. In this review the aim of the present paper is to critically discuss the male phenotype associated with low PRL in light of subsequent studies including also meta-analytic results.

## Methods

A comprehensive review was performed using Medline, Embase, and Cochrane searches, including the following words: (“hypoprolactinaemia“[All Fields] OR “hypoprolactinemia“[All Fields]) AND ((male[Filter]) AND (english[Filter])). Publications from January 1, 1969, up to March, 31st, 2024 were included. In addition, to better analyze the relationship between low PRL and metabolic derangements, data obtained with the latter search were used for a meta-analytic approach. In particular, a meta-analytic approach was selected in order to minimize possible sources of bias derived from a personal interpretation of the data. Meta-analysis was performed using Comprehensive Meta-analysis Version 2, Biostat, and (Englewood, NJ, USA).

### Hypoprolactinemia and sexual dysfunction

**Fig. 1:**
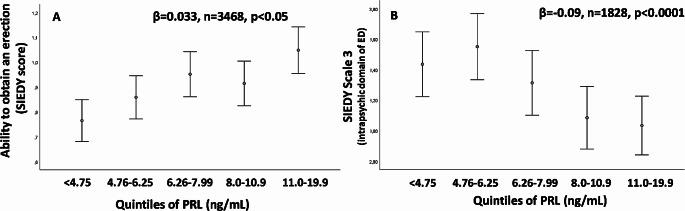
Relationships between prolactin (PRL) within the range of normal levels and ability to obtain an erection (as derived from Structured Interview on Erectile Dysfunction, SIEDY scale, Panel **A**) or the intrapsychic domain of erectile dysfunction (SIEDY Scale 3, Panel **B**). The label reports the association according to a linear regression model using log transformed PRL levels as continuous variable. The figure reports PRL divided into quintiles for graphical purposes. The relationships retain significance after adjusting for age, lifestyle (smoking and drinking behavior), body mass index (BMI) and testosterone levels

In our aforementioned first original description [10], subjects in the lowest PRL quartile showed a higher prevalence of ED and reduced penile blood flow, as measured by penile color Doppler ultrasound (CDU) after prostaglandin E1 (PGE1) stimulation. Later on, these data were confirmed in an extended cohort of the previous one, demonstrating that men with moderate or severe ED have lower levels of PRL than those with milder forms of ED [11]. In another cross-sectional study [12], involving a cohort of 2,948 European community-dwelling men aged 40–79 years old (European Male Aging Study, EMAS), lower levels of PRL were associated with worsening sexual functioning as compared to the previous year, as measured by a specific domain (Change in Sexual Function, CSF) of the EMAS Sexual Function Questionnaire (ESF) [13]. In particular, as compared to the previous year, subjects with the lowest PRL levels reported more often a decrease in spontaneous and sex-related erections [12]. Figure 1, panel A, shows the relationship between PRL levels within the range of normality (< 20 ng/ml, represented as quintiles) and the ability to obtain an erection sufficient to penetrate the partner (as derived from the Structured Interview on Erectile Function, SIDEY score, [14]) in a large series of subjects consulting for sexual dysfunction. After adjusting for age, lifestyle (smoking and drinking behavior), body mass index (BMI), and testosterone levels, PRL (log-transformed) was directly associated with a better erection. We previously reported [14] and validated [14–16] a multidimensional structured interview (SIEDY) able to provide scores on the different pathogenetic domains underlying ED, including an organic component (Scale 1), a relational component (Scale 2), and an intrapsychic component (Scale 3). Interestingly, PRL level within the physiological range, was not associated with Scales 1 and 2, but it was with Scale 3 (Fig. 1, panel B). The latter observation suggests that lower PRL levels are more often associated with a psychogenic form of ED. How reduced PRL levels might be more prevalent in patients with psychogenic ED is a matter of speculation. The central nervous system is endowed with PRLRs, which regulate several functions, including parental and sexual behavior [17]. A single (10 micrograms) administration of ovine PRL in the rat facilitated sexual activity and increased extracellular striatal metabolites of dopamine and serotonin (5-HT), while chronic administration sorted the opposite Fig. (18). Male rats with normal mating activity showed enhanced sexual behavior when injected subcutaneously with rat PRL (5, 10–50 µg/kg). In animals with poor sexual performance or in impotent rats, PRL (5–10 µg/kg, but not 50 µg/kg) restored the full pattern of sexual behavior [19]. In twelve heterosexual men, basal PRL levels were positively associated with sexual response-related brain activity during visual sexual stimulation, as recorded by functional MRI [20]. Hence, central PRL might facilitate the acute response to a sexual stimulus. However, it is also possible that PRL, having a central anti-anxiety activity ( [21], see also below), could facilitate penile erection by decreasing performing anxiety. This observation is also in line with the negative relationship between PRL levels and SIEDY Scale 3, i.e., psychogenic ED (see Fig. 1, Panel B; see below).

In the original report on hypoprolactinemia [10], we also observed a negative association between PRL levels and the propensity to ejaculate, which was confirmed later on [11, 22–24]. In the aforementioned EMAS study [12], it was observed that men reporting a severe reduction in the ability to reach orgasm, as compared to the previous year, showed lower PRL levels. Hence, PRL might facilitate the orgasmic reaction and ejaculation. In line with this hypothesis, in a study on 288 men with couple infertility, we observed a positive relationship between PRL levels and ejaculate volume, but not with other parameters of the seminal analysis [24]. Considering that the large majority of the ejaculate volume is derived from the prostate and seminal vesicles, we hypothesized a relationship between PRL levels and male accessory glands. We found that men in the lowest PRL quartile showed a reduced seminal vesicle (SV) volume either before or after ejaculation at color doppler ultrasound (CDU) and reported a lower ability to control ejaculation [24], as assessed by a specific questionnaire (Premature Ejaculation Diagnostic Tool; PEDT, [25]). These findings were in keeping with old observations on the trophic effect of PRL on male accessory glands. In one of the first experiments, hyperprolactinemia, induced by grafting a rat anterior pituitary under the kidney capsule, was associated with an almost double SV size, which is androgen-independent [26]. Interestingly, targeted disruption of the PRL gene induced SV and prostate hypotrophy, although fertility was preserved [27]. Accordingly, expression of PRL in different modes of transgenic mice was associated with prostate hypertrophy [28]. Besides the hypothesis of a trophic effect of PRL a male accessory gland that can peripherally regulate the orgasmic platform, it is possible that circulating PRL might mirror the central activity of the serotoninergic system. In fact, serotonin (5-HT) is the neurotransmitter more deeply involved in controlling ejaculation and is also a PRL-releasing factor [11]. Accordingly, 5-HT reuptake inhibitors (SSRI) are considered the first-line treatment for premature ejaculation and their use is associated with a PRL increase [29–31]. In a study in rhesus monkeys, it was found that the concentration of 5-hydroxyindolacetic acid (the major 5-HT metabolite) within cerebrospinal fluid correlated tightly with salivary PRL concentration, whereas other monoamine metabolites did not [32]. In that study, it was postulated to use peripheral PRL as a surrogate marker of central 5-HT turnover [32].

### Hypoprolactinemia and psychological disturbances

PRL is considered a stress-related hormone [21]. Physiologically, its elevation during pregnancy and puerperium might help mothers to be more resilient towards perinatal stressful conditions, including nursing and breastfeeding, allowing the young mother to enable reproductive success. In the rat, chronic intracerebroventricular infusion of antisense oligonucleotides against the PRLR increased anxiety and depressed maternal behavior, which was associated with increased ACTH levels [33]. Hence, hyperprolactinemia, a well-recognized natural and beneficial phenomenon during pregnancy and lactation, could be helpful even in smoothing related anxiety (reviewed in [34]).

**Fig. 2:**
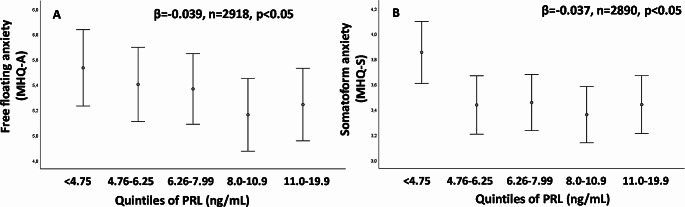
Relationships between prolactin (PRL) levels within the range of normality (< 20 ng/ml, represented as quintiles) and free-floating anxiety (as derived from Middlesex Hospital Questionnaire, MHQ-A score, Panel **A**) or somatoform anxiety (MHQ-S, Panel **B**). The label reports the association according to a linear regression model using log transformed PRL levels as continuous variable. The relationships retain significance after adjusting for age and the use of psychotropic medications

It is interesting to note that since the first description of the hypoprolactinemic phenotype, we have reported higher levels of free-floating anxiety in subjects in the lowest quartiles of PRL levels [11]. Figure 2, panel A, confirms, in a larger cohort of subjects with sexual dysfunction, the dose-dependent association. The relationship was confirmed after adjusting for age and the use of psychotropic medications (Fig. 2, Panel A). In a similar adjusted model, we now originally report an association between low PRL and higher levels of somatizing anxiety (Fig. 2, Panel B). Interestingly, free-floating anxiety was often observed in patients with premature ejaculation [35] and somatizing anxiety in patients reporting erectile dysfunction [36, 37]. In the EMAS study [12], we observed that low PRL was more often present in those reporting increased numbers of adverse life events in the previous six months. In addition, in the same study [12], we found that in subjects with overt depressive symptomatology (high BDI) scores, the severity of symptoms was higher in those with the lowest level of PRL. In 212 Italian families phenotyped for type 2 diabetes mellitus (T2DM) and major depressive disorders, an association between T2DM and variation in the PRLR gene was found, suggesting a mental-metabolic role for PRL [38]. PRL response to a serotoninergic stimulus (fenfluramine or citalopram) has been extensively used to evaluate central serotonergic function in affective and related disorders [39–41]. In depressive subjects, the acute response of PRL to a challenge with these agents was significantly blunted [39–41]. It has been recently shown that the stimulatory effect of 5-HT on PRL release is due to hyperpolarization and abolishing of phasic discharge in rat neuroendocrine tuberoinfundibular dopamine (TIDA) neurons by using in vitro whole-cell patch-clamp recordings [42]. All the aforementioned findings [32, 39–42] suggest that circulating PRL could be considered a mirror of serotoninergic activity in the brain, as previously hypothesized [10–12]. This hypothesis is also in keeping with the relationship between low PRL and premature ejaculation because 5-HT activity is the main controller of time to ejaculation [23]. Given the well-known role of brain 5-HT in anxiety and depressive symptoms [43], we are tempted to speculate that looking at peripheral PRL levels might give insights into the central activity of the serotoninergic system and related psychological functioning.

### Hypoprolactinemia and metabolic derangements

**Fig. 3:**
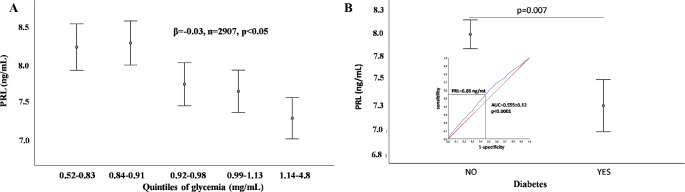
Panel **A**: Relationship between fasting glucose and prolactin (PRL) levels. The label reports the association according to a linear regression model using glycemia as continuous variable. The figure reports glycemia divided into quintiles for graphical purposes. The relationship retains significance after adjusting for age, lifestyle (smoking and drinking behavior), use of psychotropic medications, chronic disease score (a broader index of morbidities), and TSH. Panel **B**: PRL levels in diabetic and non-diabetic subjects. The relationship retains significance after adjusting for age, lifestyle (smoking and drinking behavior), use of psychotropic medications, chronic disease score (a broader index of morbidities), and TSH. Inset of panel B: ROC curve analysis for PRL levels in discriminating diabetic and non-diabetic men. The value of PRL 6.86 ng/mL represents the best threshold level below which diabetic and non-diabetic men may be distinguished with the best operating characteristics

A negative relationship between PRL (within the reference range) and blood glucose was originally described by us 15 years ago in a study on patients consulting for sexual dysfunction [10]. In that study, we also found that patients in the lowest quartile of PRL showed the highest prevalence of T2DM [10]. These findings were later confirmed in the EMAS cohort [12]. Figure 3, panel A, shows the negative relationship between PRL and glycemia in 2907 subjects consulting for sexual dysfunction, with normal PRL levels. The relationship retains significance after adjusting for age, lifestyle (smoking and drinking behavior), use of psychotropic medications, chronic disease score (a broader index of morbidities), and thyroid-stimulating hormone (TSH) levels. The same figure, panel B, reports PRL levels in men of the same cohort having or not having any form of diabetes mellitus (DM), after adjusting for the aforementioned confounders. A fully-adjusted binary regression model indicates that for each 0.5 ng/ml of PRL increase the risk of DM decreases by almost 2% (OR = 0.981 CI = 0.966;0.995). Figure 3, Panel B inset, also reports the ROC curve for PRL in distinguishing men having or not having any form of DM. At PRL values lower than 7 ng/ml, there is a 55% sensitivity and specificity for having DM. At the same PRL threshold, the corresponding positive and negative predictive values are 56.9 and 51.9% respectively. Hence, although with a rather low specificity and sensibility, low PRL is a significant correlate of DM. In previous studies, we also described an association between low PRL, dyslipidemia, and the construct of metabolic syndrome (MetS), as defined by National Cholesterol Education Program Expert Panel (NCEP) criteria [10–12]. In a study of 345 volunteers, it was reported that the PRL response to the serotoninergic agent citalopram was decreased in those satisfying the NCEP criteria for MetS [44] and similar results were reported in another cohort with the fenfluramine-induced PRL increase [45]. Later on, the same group reported that decreased PRL responsiveness to citalopram was associated with a greater intima-media thickness of the carotid artery, a broader index of preclinical atherosclerosis [46]. More recently, low PRL was demonstrated as a valid tool to predict non-alcoholic fatty liver disease [47]. All these findings suggest that low PRL is associated with a poor metabolic phenotype. This view is further substantiated by two recent meta-analyses of epidemiological studies suggesting that low PRL is associated with a higher risk of T2DM in men and women [6, 48]. We now report an updated meta-analysis of epidemiological studies focusing only on the metabolic features of men.

Cross-sectional data comparing male subjects with or without reduced levels of PRL were present in 10 studies [10, 12, 49–56]. The characteristics of the retrieved trials and the types of outcomes considered are reported in Table 1. Overall, 7007 patients were included, with a mean age, baseline PRL, and BMI of 52.3 years, 8.90 ng/ml, and 26.5 kg/m^2^, respectively. The mean, considering the lowest PRL levels, was 7.06 ng/ml (Table 1). The criteria used for the definition of low PRL differ among the studies (Table 1).

**Table 1:** Characteristics of trials included in the study. BMI = body mass index; PRL = prolactin; * only men were considered. Lowest PRL level refers to the lowest threshold considered in the included studies

Study	*N*°	Mean age (years)	BMI (Kg/m^2^)	Type of population	Mean PRL levels (ng/ml)	Reduced PRL definition	Lowest PRL levels (ng/ml)
*Cross-sectional studies*							
Corona et al., 2009 [10]	1040	52	313	Erectile dysfunction	7.47	I vs. IV quartile	5.31
Reuwer et al., 2009* [49]	1004	64.5	26.7	Population based	9.88	I vs. III tertile	8.39
Ballach et al., 2013* [50]	1966	52.1	27.3	Population based	4.84	I vs. IV quartile	-
Wang et al., 2013 [51]	1034	60	25.1	Population based	8.69	I vs. IV quartile	6.32
Corona et al., 2014 [12]	1448	60	27.8	Population based	8.49	I vs. IV quartile	5,63
Wang et al., 2016* [52]	309	60.5	24.9	Population based	8.46	I vs. IV quartile	6.51
Chahar et al., 2017* [53]	90	52.8	24.4	People presenting for check-up at medicine outpatient facility	9.68	I vs. IV quartile	7.11
Ruiz-Herrera et al., 2017 [54]	27	37.7	27.2	Surgery involving access to the abdominal cavity	11.96	<249 mU/L	11.7
Jayashankar et al., 2022 [55]	64	43.4	26.8	Type 2 diabetic outpatients	11.07	I vs. IV quartile	7.11
Sheoran et al., 2023 [56]	25	-	27.9	Type 2 diabetic outpatients	7.99	I vs. IV quartile	5.10
*Longitudinal studies*							
Ballach et al., 2013* [50]	1966	52.1	27.3	Population based	4.83	I vs. IV quartile	-
Wang et al., 2016* [52]	309	60.5	24.9	Population based	8.46	I vs. IV quartile	6.51

**Fig. 4:**
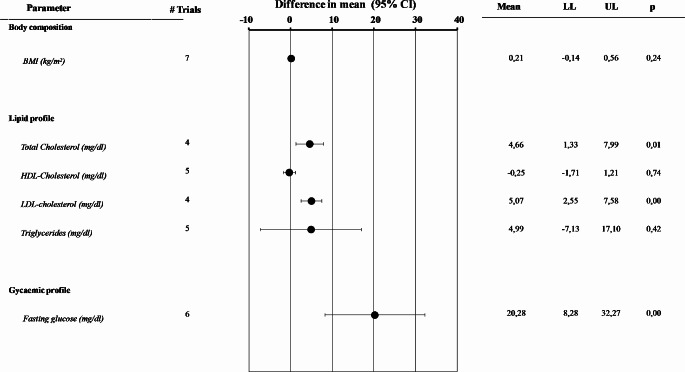
Overall differences in several body composition and glycometabolic parameters in patients with against those without reduced prolactin levels. BMI = body mass index; HDL = high density lipoprotein; LDL = low density lipoprotein. LL = lower levels; UP = upper levels

**Fig. 5:**
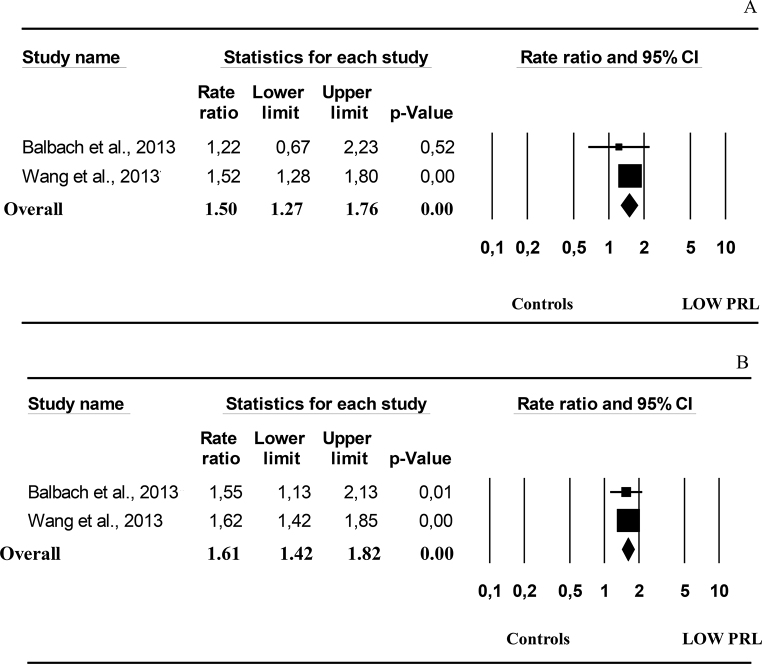
Age-adjusted (**A**) and fully-adjusted (**B**) risk for diabetes mellitus as derived from cross-sectional data. LL = lower levels; UP = upper levels

I^2^ for BMI related to cross-sectional data was 41.2, *p* = 0.116. The funnel plot and Begg-adjusted rank correlation test suggested no major publication bias (Kendall’s τ: 0.764; *p* = 0.095). No differences in BMI were observed when subjects with low PRL (LOW PRL) were compared to controls (Fig. 4 and Supplementary Fig. 1, Panel A). However, subjects with LOW PRL, showed elevated total and LDL cholesterol (Fig. 4 and Supplementary Fig. 1, Panels B and C), but no differences in HDL cholesterol and triglyceride levels when compared to controls (Fig. 4 and Supplementary Fig. 1, Panel D and E). In addition, higher fasting glucose levels were observed in patients with LOW PRL when compared to controls (Fig. 4 and Supplementary Fig. 1, Panel F). In line with these data, the age-adjusted and fully-adjusted risk of having DM, was increased in subjects with LOW PRL when compared to controls (Fig. 5, Panels A and B). Although the criteria used to define low PRL and the characteristics of the control groups differ among studies, this meta-analysis, in keeping with previous ones in both genders [6, 48], suggests that low PRL is a positive correlate for a worse metabolic profile and DM, although the magnitude of the PRL effect is quite low.

The direction of the association between low PRL and the risk of a worse metabolic profile and DM is a matter of debate. Preclinical studies suggest that PRL has a trophic effect on pancreatic β cells, glucose entry into the β cells, and glucose-stimulated insulin secretion (reviewed elsewhere [4, 48]). PRLR-deficient mice had 26–42% reductions in islet density and beta-cell mass, along with a reduced insulin content and a reduced insulin response to a glucose load [57]. Most probably, these effects of PRL on the pancreas are related to the maternal energetic adaptation to pregnancy. Accordingly, targeted deletion of PRLR in the mouse β cells is associated with elevated blood glucose and gestational diabetes [58]. Data in humans suggest that PRL in the “normal” range (7-100 ng/mL, as defined in [5]) shows a beneficial effect on metabolism by increasing adiponectin levels, reducing insulin resistance, and regulating healthy visceral fat expansion (reviewed in [4, 5, 59]). We here provided evidence that indeed, PRL levels below 7 ng/mL are associated with a higher prevalence of DM, even though the relative specificity and sensitivity were rather low (inset Fig. 3 Panel B). Similar results were recently confirmed in the EMAS study [60]. Longitudinal studies can help in understanding if a lower PRL level at baseline can favor a forthcoming diabetic state at follow-up. A previous meta-analysis of the few (*n* = 3) available studies indicates that this is not the case, although there is a tendency in women [48]. In contrast, a more updated meta-analysis [6] including both genders found an association between the age-adjusted risk of DM and low PRL (OR = 1.3 [1.11–1.52], *p* < 0.0001). However, when the analysis was restricted to men, statistical significance was lost, most probably because of the limited number of observations available (*n* = 2, OR = 1,03[0.65;1.65]; *p* = 0.90).

It is also possible that a decrease in PRL is a response to an overload of nutrients. A glucose load during the oral glucose tolerance test (OGTT) was associated with a decline in PRL levels more evident in control than in type 1 diabetic subjects [61]. Interestingly, in a study on a large cohort of male and female individuals with normal PRL levels, the area under the curve (AUC) of OGTT was positively associated with lower PRL levels [62]. In another study, the PRL response to citalopram was smoothed by an acute load of lipids [63]. Hence, reduced PRL levels can only be a consequence of an unhealthy lifestyle, characterized by an overload of food with an elevated glucidic and lipidic content.

Other working hypotheses deal with the possibility that low circulating PRL just reflects increased or decreased dopaminergic and serotoninergic activity, respectively, within the central nervous system (CNS). An increased dopaminergic activity that favors impaired glucose metabolism (see in [59] for review) is counterintuitive, because bromocriptine, a dopaminergic agonist, has even been approved for the treatment of DM since 2009. In addition, the clinical use of several dopaminergic antagonists (antipsychotics) is associated with impaired metabolism, metabolic syndrome, and DM [64]. As stated before and previously published [10–12], the serotoninergic hypothesis seems the most reasonable, i.e., low PRL is just a byproduct that reflects a decreased serotoninergic activity within the CNS, as demonstrated in apes [32]. Considering that low serotoninergic activity is still the major hypothesis underlying the pathogenesis of anxiety and depression [65, 66], it is interesting to note that depressed people have a 41% increased risk of developing DM and a 32% increased risk of developing T2DM in a meta-analysis [67]. Although the serotoninergic hypothesis explaining the biology of anxiety and depression is under debate [66, 67], it is a matter of fact that serotoninergic medication still represents a valuable treatment for anxiety and depression [68], and, by the way, most of them increase PRL levels [11, 30].

Considering the worse metabolic profile associated with low PRL, it is possible to speculate that those with the highest PRL values, but within the normal range, may have lower CV risk in longitudinal studies. In a previous study on subjects with ED (therefore at high CV risk), we reported that, in both unadjusted and adjusted analyses, there was a lower incidence of major CV events (MACE) in subjects with PRL levels in the highest PRL quintile when compared with the rest of the sample [69]. In the population-based Study of Health in Pomerania (SHIP), it was found that low PRL in men was associated with increased left ventricular mass and with incident left ventricular hypertrophy, indicating greater cardiac remodeling [70]. However, results from the same SHIP cohort, published later on [71], did not show an increased CV mortality in men with lower PRL levels, but even the reverse. A nested case–control study in the EPIC-Norfolk cohort showed no association between higher serum PRL levels and incident coronary artery disease among healthy men and women aged 40–79 who were followed up for an average of 7 years [49]. Finally, in the Framingham Heart Study, involving men and women who attended two examinations an average of 6.1 years apart, no relationship was observed between PRL levels and CV diseases [72]. Hence, the relationship between normal PRL levels and CV diseases and related mortality is still a matter of debate [7]. Considering that even the hyperprolactinemic condition is associated with obesity, insulin resistance, dyslipidemia and hyperglycemia (reviewed in [4–6]) - most probably reflecting the physiological adaptation to pregnancy - the aforementioned contrasting results can be explained.

## Conclusions

The hypoprolactinemic male phenotype here described is characterized by (i) a worse metabolic phenotype (including DM), (ii) increased psychological disturbances (including anxiety and depression), and (iii) sexual dysfunctions (including psychogenic ED and ejaculatory dysfunctions). These features may be the result of some deficiency in the pleiotropic action of PRL in the peripheral tissues (pancreas, adipose tissue, male accessory glands) or within the CNS. In all these tissues, PRLR was described (reviewed in [4–6, 28]). If this is the case, (over)treatment with dopaminergic medications should induce metabolic derangements, whereas a meta-analysis of trials in prolactinoma demonstrated that they reverse metabolic abnormalities [6]. In addition, in meta-analyses, dopamine agonists in T2DM significantly lowered fasting glucose and triglyceride levels, along with HbA1c, without causing severe negative effects, including CV events [73, 74]. Finally, elevating PRL levels with antidopaminergic medications is not associated with a more favorable metabolic profile, which is even worsened [64]. This evidence argues against the direct role of PRL in the hypoprolactinemic phenotype. Hence, low PRL, at the present time, cannot be considered an useful predictive marker for forthcoming T2DM.

An alternative view is that low PRL is not the cause but the consequence of conditions associated with the described phenotype. We originally hypothesized that low PRL is a mirror of events associated with a decrease in serotoninergic tone in the CNS [10–12]. Along with TRH, 5-HT is one of the main releasers of PRL at the hypothalamic level, having a negative action on tuberoinfundibular dopaminergic neurons [42]. A decrease in serotoninergic activity is by far associated with anxiety and mood disturbances [43, 66], which, are all improved by treatment with serotoninergic agents [67]. Similarly, decreased serotoninergic activity is associated with ejaculatory dysfunctions, including premature ejaculation (PE). Serotoninergic medications are considered the first-line treatment for PE [23]. The higher prevalence of psychogenic ED in hypoprolactinemia [10–12] could be the result of the increased level of anxiety, particularly somatoform anxiety [36]. Finally, mood disturbances and decreased central serotoninergic activity per se can be the cause of the worse metabolic profile here described. In the serotoninergic dorsal raphe nucleus, increased activity reduces food intake, while a reduction in 5-HT release increases food intake through a complex interaction with GABA and glutamatergic neurons ( [75, 76] see in [77] for review).

Some limitations to our view of the hypoprolactinemic male phenotype should be recognized. Our view is based mostly on results from a few centers and, in particular, mostly on subjects complaining of sexual dysfunction. In addition, all the associations characterizing the male hypoprolactinemic phenotype here described are of relatively weak magnitude, suggesting that, overall, low PRL has only an ancillary role in their determinism. However, we here propose that, as for other hormones, a deficiency of PRL could suggest to health care professionals an underlying phenotype that needs to be further investigated.

## Electronic supplementary material

Below is the link to the electronic supplementary material.

Supplementary Material 1

## Data Availability

The datasets used during the current study are available from the corresponding author on reasonable request.
