# Supplementary material for: Acquired hypoprolactinemia in men, possible phenotype
Source: Rev Endocr Metab Disord. 2024 Jul 27;25(6):1109–19. doi: 10.1007/s11154-024-09895-9 (PMC11624216; doi:10.1007/s11154-024-09895-9)
Supplement: Supplementary file 1 — Supplementary Material 1 [file 11154_2024_9895_MOESM1_ESM.docx]

**Supplementary Figure 1**. Weighted mean differences (with 95%CI) between patients with reduced prolactin levels (LOW PRL) and controls on several parameters including body mass index (A); total cholesterol (B), low density lipoprotein cholesterol (C), triglycerides (D), high density lipoprotein cholesterol (E), and fasting glucose (F). LL=lower levels; UP=upper levels. *only men; ** chronic kidney disease.
